# Prospecting Cellular Gold Nanoparticle Biomineralization as a Viable Alternative to Prefabricated Gold Nanoparticles

**DOI:** 10.1002/advs.202105957

**Published:** 2022-05-04

**Authors:** Aaron S. Schwartz‐Duval, Konstantin V. Sokolov

**Affiliations:** ^1^ Department of Imaging Physics The University of Texas MD Anderson Cancer Center 1515 Holcombe Boulevard Houston TX 77030 USA; ^2^ The University of Texas MD Anderson Cancer Center UTHealth Graduate School of Biomedical Sciences 6767 Bertner Ave Houston TX 77030 USA; ^3^ Department of Bioengineering Rice University 6100 Main St. Houston TX 77030 USA; ^4^ Department of Biomedical Engineering The University of Texas at Austin 107 W Dean Keeton St. Austin TX 78712 USA

**Keywords:** bioimaging, biomineralization, gold nanoparticles, in situ therapy, theragnostic

## Abstract

Gold nanoparticles (GNPs) have shown considerable potential in a vast number of biomedical applications. However, currently there are no clinically approved injectable GNP formulations. Conversely, gold salts have been used in the clinic for nearly a century. Further, there is evidence of GNP formation in patients treated with gold salts (i.e., chrysiasis). Recent reports evaluating this phenomenon in human cells and in murine models indicate that the use of gold ions for in situ formation of theranostic GNPs could greatly improve the delivery within dense biological tissues, increase efficiency of intracellular gold uptake, and specificity of GNP formation within cancer cells. These attributes in combination with safe clinical application of gold salts make this process a viable strategy for clinical translation. Here, the first summary of the current knowledge related to GNP biomineralization in mammalian cells is provided along with critical assessment of potential biomedical applications of this newly emergent field.

## Graphical abstract

Mammalian cells can synthesize nanoparticles from ions from gold salts. Gold salt drugs have been clinically applied for nearly a century, with evidence of nanoparticle formation within those patients. However, the mainstream use of these gold drugs considerably predates popular study of gold particles. Here a newly emergent field is summarized, wherein biomedically relevant gold particles are prepared in situ.

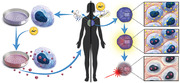

## 1 Introduction

Throughout history and prehistory, humankind has been fascinated with the possibility of medical applications of gold.^[^ ^1^ ^]^ This fascination has continued into modern times; in the past three decades, gold nanoparticles (GNPs) have been a ubiquitous staple in biomedical nanoparticle research. With more than 100 000 peer‐reviewed studies published on the subject of GNPs since 1995, the global communal knowledge and demonstrated potential of GNPs for biomedical applications are vast. GNPs have been touted for their tunable optical properties related to their size^[^ ^2^ ^]^ and shape^[^ ^3^ ^]^ and for ease of surface functionalization.^[^ ^4^ ^]^ These properties have been the source of much success in the development of in vitro diagnostics.^[^ ^5^ ^]^ Owing to the highly reactive and relatively simplistic nature of the favored precursor for GNP synthesis—chloroauric acid (HAuCl_4_)‐ virtually any nanoscale size or shape could be achieved, and the synthesis was shown to be reproducible by multiple laboratories.

Once formed, GNPs are stable and largely considered bioinert,^[^ ^6^ ^]^ enabling their use in biological settings. The “bioinertness” of GNPs, tunable morphology, and morphologically dependent optical properties, combined with the high‐Z number of gold atoms that is beneficial in interactions with x‐rays led to many successful preclinical therapeutic and diagnostic studies. For therapeutics, GNPs can act as radiosensitization agents for radiation therapy,^[^ ^7^ ^]^ and as vehicles for the conversion of electromagnetic energy to thermal energy for photothermal ablation.^[^ ^8^ ^]^ For diagnostics, GNPs are able to provide contrast for computed tomography (CT),^[^ ^9^ ^]^ optical,^[^ ^10^ ^]^ and photoacoustic imaging.^[^ ^11^ ^]^ However, thus far, there are no clinically approved parenteral GNP applications,^[^ ^12^ ^]^ with only few reaching clinical trials.^[^ ^13^ ^]^

Unlike GNPs, gold salts have been used in the clinic in the treatment of rheumatoid arthritis for nearly a century.^[^ ^14^ ^]^ Although these gold salt treatments are not as prevalent in the clinic today as previously, since more effective drugs are now available, gold salts are being reappropriated and investigated for applications against other diseases (e.g., cancer, HIV, bronchial asthma, and malaria).^[^ ^15^ ^]^ These past clinical applications of gold salts also provided evidence of nanoparticle formation within treated patients.^[^ ^16^ ^]^ However, gold as a nanoparticle colloid with its potential applications was not fully appreciated at that time and thus was not taken advantage of.

In this review, we evaluate strategies reliant on the application of gold salts that can intentionally enable the biomineralization of GNPs in situ as a viable, translatable alternative or complement to the classic nanomedicine approach, wherein nanoparticles are prepared through benchtop syntheses and then administered to patients. This approach, wherein particles are generated on‐site may have significant clinical potential for improving transport within dense biological environments because it changes the current delivery methodology from pre‐made GNPs with sizes of 5–200 nm to delivery of ≈0.3 nm gold ions (≈16 to 1400‐fold size reduction), and formed particles may even be packaged within exosomes. These strategies take advantage of the long history of the application of gold salts and the vast wealth of knowledge and demonstrated potential of GNPs in biomedical applications, while making use of the biological machinery to drive gold biomineralization‐based theranostics. We acknowledge the potential for in situ nanoparticle applications other than gold, and also with non‐mammalian cell‐based biosynthesis. Microbial gold biosynthesis is generally better understood than biomineralization by mammalian cells,^[^ ^17^ ^]^ and there are many studies showing exciting results for biomedical applications of biomineralization or in situ nanoparticle formation from other materials as well.^[^ ^18^ ^]^ Herein, we choose to focus primarily on biomineralization of GNPs by mammalian cells for this review because of how it combines and intertwines three related fields that each have significant long‐term background research but with seemingly little overlap, namely, 1) GNPs in medicine,^[^ ^1^, ^2^, ^3^, ^4^, ^5^, ^6^, ^7^, ^8^, ^9^, ^10^, ^11^, ^13^ ^]^ 2) geobiological cycling of heavy metals by microbes,^[^ ^17^ ^]^ and 3) chrysotherapeutics.^[^ ^14^, ^15^, ^16^, ^19^ ^]^

## 2 Chrysotherapy

**Figure 1:**
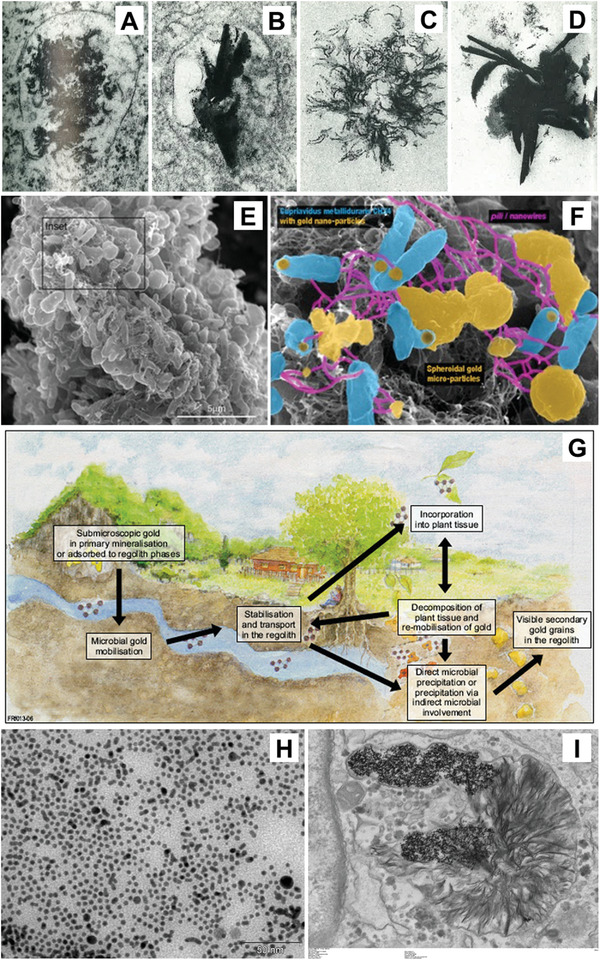
Biological cycling of gold. A–D) TEM images of aurosomes resulting from treatments of gold salts. Reproduced with permission.^[^ ^19i^ ^]^ Copyright 1981, Elsevier. E) Schematic of the geobiological cycle of gold with scanning electron microscopy of *Cupriavidus metallidurans* biofilm with highlight of nanoparticle‐cell associations. E–G) Reproduced under the terms of the Creative Commons CC‐BY license.^[^ ^17f^ ^]^ Copyright 2013, The Authors. Licensee by MDPI. H) TEM of GNPs applied to primary human fibroblasts and I) the cells after 2 weeks of incubation. Reproduced under the terms of the Creative Commons CC‐BY license.^[^ ^27b^ ^]^ Copyright 2019, National Academy of Science.

Gold salts were used in clinical settings since the late 1920s in the treatment of rheumatoid arthritis.^[^ ^14^, ^19^ ^]^ These chrysotherapies (*chryso*, meaning gold) were shown to affect a number of biological processes providing antimicrobial, anti‐immunological, and anti‐inflammatory effects (i.e., interfering with microbial, immune, or inflammatory processes).^[^ ^19c^ ^]^ Although the precise mechanism of action against rheumatoid arthritis by chrysotherapies remains unclear,^[^ ^19b,c^ ^]^ some patients found relief. Chrysotherapeutic drugs are currently used less frequently for rheumatoid arthritis since more effective antirheumatic drugs are now available, but chrysotherapeutics are being investigated for their potential against other diseases including parasitic/bacterial infections, neurodegenerative disorders, AIDS, cancer, and even potentially against COVID‐19.^[^ ^19d,e^ ^]^ Just one of these gold salt drug formulations, auranofin, is being investigated in five clinical trials listed in clinicaltrials.gov. Successful clinical translation of chrysotherapies from ideation can be more easily achieved due to the historically long use in humans. Follow‐up of patients who underwent long‐term gold salt treatments suggested evidence indicative of GNP formation, namely, a blue‐purple discoloration of the skin that was clinically diagnosed as chrysiasis.^[^ ^19a,f^ ^]^ A report from 1931 showed that in 28 of 57 patients who presented with chrysiasis, skin pigmentation was dependent on the amount of gold salt (sodium aurothiosulfate) applied.^[^ ^19g^ ^]^ Patients who received larger doses of aurothiosulfate were more likely to present with chrysiasis than were those who received lower doses.^[^ ^19g^ ^]^ We can now appreciate that this pigmentation was likely due to GNP nucleation within the patient with nanoparticle deposition within their dermis. However, pigmentation alone does not necessarily indicate GNP formation. A more recent report (1989) confirmed the dermal presence of metal gold in skin biopsies of patients with chrysiasis and characterized the crystalline state of this gold.^[^ ^16^ ^]^ In this report, the authors confirmed the presence of gold by using energy dispersive x‐ray spectrometry and determined the crystallinity of the gold using electron diffraction.^[^ ^16^ ^]^ Further, transmission electron microscopy (TEM) showed crystalline nanoscaled gold particles within electron‐dense vesicular bodies termed aurosomes (**Figure** **1**A–D).^[^ ^16^ ^]^ Although this study may have been the first and only to characterize the crystallinity of gold found within aurosomes of patients with chrysiasis, aurosomes were also identified with use of all electron microscopy examinations of preclinical and clinical applications of gold salts regardless of formulation (i.e., aurothiosulfate, gold sodium thiomalate, aurothioglucose, or other gold‐based drugs).^[^ ^19h–k^ ^]^ This evidence suggests that crystalline nanoscaled gold particles are biomineralized in patients treated with gold salts regardless of the formulation of these salts.

## 3 Biomineralization

The term *biomineralization* encompasses biotic processes that mediate the formation of mineral compounds in conditions distinct from those of inorganic mineralization (i.e., high‐pressure and high‐temperature mineralization conditions).^[^ ^20^ ^]^ In the biomedical realm, the majority of biomineralization focuses on calcium with bone development, as well as the formation of kidney and salivary stones,^[^ ^21^ ^]^ with some intriguing calcium biomineralization strategies for therapy.^[^ ^22^ ^]^ However, biomineralization is an extremely widespread process that occurs in all taxonomic kingdoms.^[^ ^23^ ^]^ In humans, a wide variety of minerals are considered essential for good health (e.g., calcium, chromium, copper, iron, magnesium, manganese, molybdenum, potassium, sodium, and zinc).^[^ ^24^ ^]^ These minerals enable many biological processes that have been conserved since soon after life began on Earth.^[^ ^23^ ^]^ Considering nanoparticle biomineralization, the classic example is found through magnetotactic bacteria,^[^ ^25^ ^]^ named for their ability to move with directional guidance from Earth's magnetic field. This ability is granted from the unique organelles inside these bacteria—*magnetosomes*—containing magnetite (Fe_3_O_4_) nanoparticles. Biomineralized iron oxide for magnetosensation has been also found in many animals, including birds and bees, and some evidence suggests that magnetoreception exists for humans as well.^[^ ^26^ ^]^ What About Gold?

The biological cycling of gold is well established in the geoscience community, wherein microorganisms have been shown to be responsible for the cyclic mobilization and concentration of submicroscopic primary grains of gold to secondary macroscopic grains (Figure 1E–G), more commonly known as *nuggets*.^[^ ^17f^ ^]^ Currently, biomining strategies that are reliant on these microorganisms to extract metal from ore are largely seen as niche or green alternatives to conventional techniques,^[^ ^17g,h^ ^]^ but strategies such as these were used since before knowledge of microorganisms even existed, as early as the first century BC by ancient Romans.^[^ ^17g,i^ ^]^ Furthermore, there is mounting evidence that GNPs are not as inert in mammalian biological systems as previously believed.^[^ ^27^ ^]^ Recent reports have shown evidence for the biological cycling of GNPs in vivo within mice^[^ ^27a^ ^]^ and in vitro in primary human fibroblast cells.^[^ ^27b^ ^]^

In 2015, Kolosnjaj‐Tabi et al.^[^ ^27a^ ^]^ explored the long‐term (1 year) fate of gold/iron oxide nanoparticle heterostructures in mice. From this study,^[^ ^27a^ ^]^ the authors found evidence for a two‐stage degradation process, with primary dissolution of iron crystals followed by a secondary degradation and reformation of gold particles. More recently, the same group explored degradation and reformation of GNPs lacking any iron oxide component in a primary human fibroblast cell culture.^[^ ^27b^ ^]^ In this study, the authors found that aurosome‐like structures, similar to those found in chrysiasis patients, formed in cells treated with GNPs after 1 week (Figure 1H,I).^[^ ^27b^ ^]^ The authors found that the nanoparticles in the aurosomal structures had a distinct size and crystallinity that differed from the nanoparticles used to treat the cells.^[^ ^27b^ ^]^ This observation suggests that biological cycling of gold can occur in human cells wherein nanoparticles can be oxidized to ionic form and recrystallized to nanoparticles intracellularly. In a review from this same group,^[^ ^27c^ ^]^ the authors suggested that larger GNPs can be recrystallized from gold‐containing aurosome structures. Below, we discuss 1) underlying principles of how GNP biomineralization can occur through processes that are innately present in mammalian cells, followed by 2) evidence of that formation, 3) environmental factors, and 4) the impact of GNP biosynthesis on mammalian cells.

## 4 Mammalian Cellular GNP Biomineralization

### 4.1 Principles of Mammalian Cell‐Based GNP Synthesis

**Figure 2:**
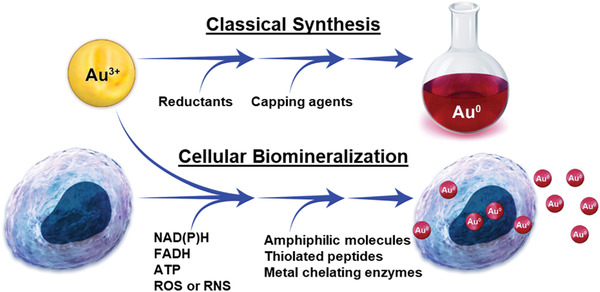
Comparison of a benchtop gold nanoparticle synthesis and gold nanoparticle synthesis through cellular biomineralization.

While the existing literature on microbial GNP biomineralization is vast, mammalian cellular biomineralization of GNPs is fairly small, with only 12 publications;^[^ ^28^ ^]^ thus, the driving factors of this process regarding mammalian cells and how it can be applied for biomedical advancement is still in very early stages of inquiry. However, GNP formation during synthesis in solution ‐ in general ‐ is well understood, initiating through a redox reaction and involving the reduction of ionic gold (typically Au^3+^ but also Au^1+^) to form Au^0^. Due to the high surface area–to–volume ratio and thus high catalytic potential of the initial gold seeds, capping agents are required to stabilize GNPs and to prevent them from forming larger crystals. In its most simple form, aqueous GNP synthesis requires at a minimum ionic gold with reducing and capping agents (**Figure** **2**). In a biological environment such as cells and tissues, all of the necessary components for GNP formation are present in the form of biomolecules with reducing and capping properties, aside from the ionic gold precursor (Figure 2). The oxidoreductive microenvironment/cellular redox potential can vary substantially, depending on cell phenotype and environment including various pathological states such as cancer. The cellular redox potential is largely determined by metabolic biomolecules such as NAD/NADH, NADP/NADPH, FAD/FADH, ATP, reactive oxygen species (ROS), and reactive nitrogen species (RNS).^[^ ^29^ ^]^ Related to capping agents, amphiphilic molecules that can spontaneously form liposomes or micelles (such as membrane lipids), thiolated molecules (such as cysteine containing proteins and peptides), and metal chelators (such as metallothioneins and metalloproteins) could potentially fill this role (Figure 2). For cellular GNP biomineralization, one should primarily consider physiologically relevant conditions of 37 ± 1 °C and a pH of 7.4 ± 0.1. When restricted to the physiological conditions, there is sufficient evidence that mammalian cells have the capacity to reduce ionic gold to a nanoparticle form;^[^ ^28^ ^]^ however, thus far, little research has been conducted on the subject so the mechanisms are still poorly understood.

### 4.2 Evidence of GNP Synthesis by Mammalian Cells

**Figure 3:**
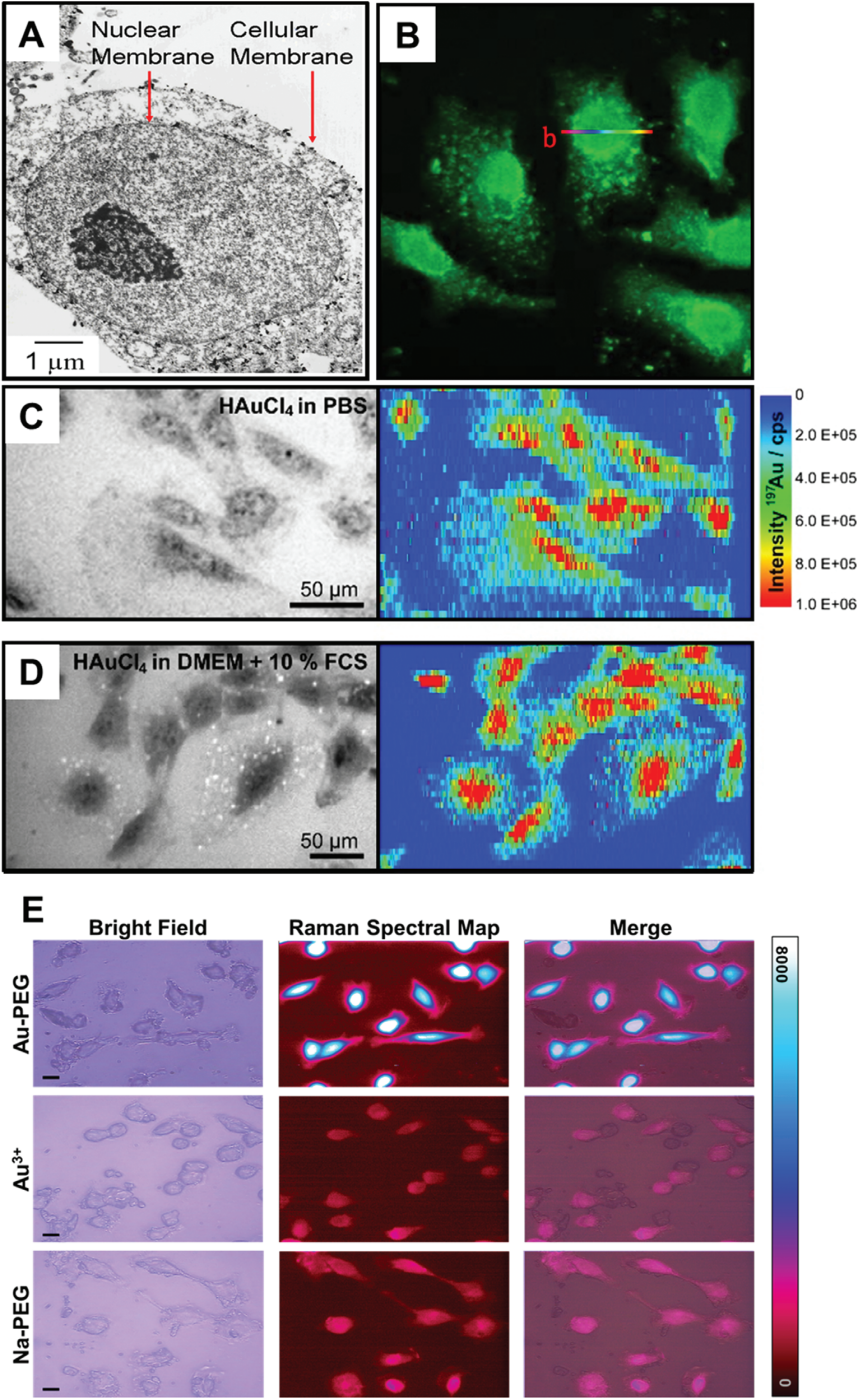
Nuclear localization of GNPs formed through biomineralization. A) TEM of GNPs formed by treating HEK 293 cells with 1 mm HAuCl_4_ in PBS for 24 h. Reproduced with permission.^[^ ^28a^ ^]^ Copyright 2005, American Chemical Society. B) Confocal fluorescence micrograph obtained using a 488 nm excitation laser of HepG2 cells incubated in full cell media supplemented with 10 µm HAuCl_4_ solutions for more than 48 h. Reproduced with permission.^[^ ^28e^ ^]^ Copyright 2013, Springer Nature. Bright field optical images and 197Au^+^ intensity distributions from LAICP‐MS of 3T3 fibroblast cells after incubation with 1 mm of HAuCl_4_ for over 24 h in either C) PBS or D) full cell media. Reproduced under the terms of the Creative Commons CC‐BY license.^[^ ^28i^ ^]^ Copyright 2017, Royal Scoeity of Chemistry. E) Bright field optical images, surface enhanced Raman spectral map, and merged bright field/Raman map images of MCF7 cells treated with either 0.24 mm Au^3+^ admixed with 10kDa hydroxyl‐terminated PEG (Au‐PEG) or 0.24 mm Na^+^ with PEG (Na‐PEG) in full cell media for 4 h. Reproduced under the terms of a Creative Commons Attribution 4.0 International License.^[^ ^28b^ ^]^ Copyright 2020, Springer Nature.

The first report of intracellular GNP formation through the application of gold salts, published in 2005 by Anshup et al., explored mammalian cells as nanoparticle “factories.”^[^ ^28a^ ^]^ In this study, human cells (kidney, HEK‐293; cervix, HeLa and SiHa; and brain, SKNSH) were treated with 1 mm HAuCl_4_ in phosphate‐buffered saline (PBS) over a 96 h period.^[^ ^28a^ ^]^ The authors used electron microscopy to visualize GNPs inside the cells (**Figure** **3**A) and observed that cellularly formed GNPs localized within the cell's cytoplasmic membrane and nucleus. This observation, wherein GNPs formed through cellular biomineralization localizing to the nucleus, would be confirmed by follow‐up reports through various methods including confocal fluorescence imaging, laser ablation inductively coupled plasma mass spectrometry (LAICP‐MS), and Raman spectral mapping (Figure 3B–E).^[^ ^28a,b,e,i^ ^]^ Also in the Anshup et al. report,^[^ ^28a^ ^]^ the authors found: 1) an increase in the plasmon resonance absorbance peak at ≈560 nm at progressing time points in supernatants of all cell types, indicating that extracellular GNP biomineralization was occurring and was increasing over time; 2) various amounts of nanoparticles in lysates of different cell types, indicating that different cell types have distinct capacities for GNP biomineralization; and 3) a greater formation of GNPs by noncancerous human embryonic kidney HEK‐293 cells compared with cancerous cervical HeLa and SiHa, as well as neuroblastoma SKNSH cells. However, contrary to this last finding by the Anshup et al.,^[^ ^28a^ ^]^ all follow‐up studies comparing cancerous and noncancerous nanoparticle biomineralization of gold found that GNP biomineralization occurs to a greater extent in cancerous cells.^[^ ^28e–h,j^ ^]^ However, these findings should not be oversimplified through cancer/noncancer comparisons, as none of the studies of GNP/GNC biomineralization within mammalian cells thus far have compared cancer/non‐cancer cells originating from the same tissue/organ type.

Later studies also found that cellular biomineralization can result in formation of fluorescent atomic gold nanoclusters (GNCs) (Figure 3B). Particles of gold that are sufficiently small (<3 nm, tens to hundreds gold atoms) can exhibit fluorescent properties, which are dependent on the number of gold atoms in each cluster.^[^ ^30^ ^]^ Typically, these GNCs are characterized by the number of gold atoms per cluster (via mass spectroscopy) rather than by the nanoparticle diameter. But what exogenous factors influence the biosynthesis of gold particles?

### 4.3 Exogenous Influences of Cellular GNP Biosynthesis

**Figure 4:**
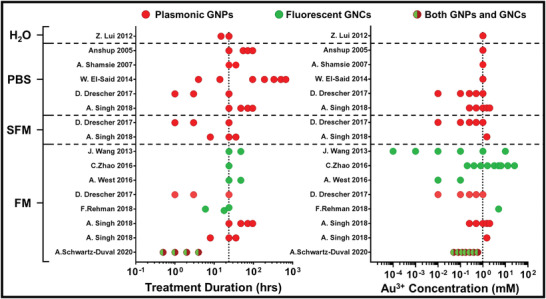
Treatment conditions during gold nanoparticle biomineralization and resulting particle optical properties. Treatment duration and concentration in relation to the observed GNPs formed through mammalian cellular biomineralization in publications with information regarding incubating media. Red or green symbols indicate observation of plasmonic GNPs or fluorescent gold atomic clusters, respectively. Combined red and green symbols indicate that both plasmonic GNPs and fluorescent gold atomic clusters were both reported. Cell incubating solutions are indicated as phosphate buffered saline (PBS), water only (H_2_O), serum free media (SFM), or full media (FM). Dotted vertical lines indicate the most common treatment conditions—24 h with 1 mm concentration of Au^3+^.

Published reports suggest that the incubation media conditions affect the cellular biomineralization of GNPs/GNCs from ionic gold. In 2017, Drescher et al.^[^ ^28i^ ^]^ explored this aspect by comparing the cellular distribution of prefabricated GNPs against GNPs formed through cellular biomineralization of Au^3+^ by 3T3/NIH cells (mouse fibroblasts) incubated in PBS, full cell media, or serum‐free (SF) cell media. To characterize intracellular distribution of gold, they used Raman spectral imaging to detect surface enhanced Raman spectra (SERS) from intracellular gold particles and LAICP‐MS to quantify total gold content within cells with a spatial resolution of 6 × 1 µm (pixel size). LAICP‐MS can determine intracellular distribution of gold regardless of its redox state. The LAICP‐MS studies showed that following the treatments with Au^3+^ ions, the majority of gold was localized inside the nucleus and in comparable amounts between treatments made in full cell media or PBS (Figure 3C,D). Whereas the cellular content of gold as quantified by LAICP‐MS was similar between treatments made in full media or PBS, this was not the case for SERS intensity. Raman signals from ionic gold treatments in PBS were 15 times greater than were those in full media. The information provided by both Raman and LAICP‐MS, indicates that gold internalization is similar between the incubating media conditions; however, plasmonic SERS active particles are more favorably produced under conditions lacking nutrients (i.e., PBS). Literature analyses indicate that fluorescent GNC production through cellular biomineralization is favored in full cell media, whereas plasmonic GNP formation is prevalent in PBS or nutrient‐free media (**Figure** **4**). However, some of these publications did not specifically focus on evaluation of both types of particle (i.e., plasmonic or fluorescent), and, therefore, they do not exclude the possibility that both GNCs and GNPs may still be present under these environmental conditions. When comparing modifications of incubation time and concentration of ionic gold treatments of cells, regarding the GNC versus GNP production via biomineralization, these relationships are not quite as clear (Figure 4).

### 4.4 Impact of GNP Biosynthesis on Affected Cells

Comparing cellular biomineralization against prefabricated 13 nm citrate‐capped GNPs, Drescher et al.^[^ ^28i^ ^]^ found that cellular GNP biomineralization resulted in two orders of magnitude higher gold intracellular content over the same treatment time period. Furthermore, for all incubation solutions (i.e., PBS, SF media, and full media), intracellular GNP synthesis resulted in prominently nuclear localization of gold, whereas cells treated with prefabricated GNPs had almost no gold within the nucleus. Drescher et al. also explored the cytotoxicity of Au^3+^ treatment on 3T3/NIH cells in full media and PBS using an XTT assay. In full media, there was no significant effect on cell viability at low HAuCl_4_ concentrations (0.01–0.10 mm); however, cell viability was reduced by more than 80% at higher concentrations (0.25–1.00 mm). From treatments made in PBS, the authors reported reduction in cell viability by more than 50% at all concentrations with an increase of ≈10% from lowest to the largest HAuCl_4_ doses (0.01 to 1.00 mm). However, this apparent increase might be explained by an overlap between the absorption spectra of the viability reporter (460 nm peak) and the GNPs formed through cellular biomineralization rather than an increase in actual cell viability. Further, Schwartz‐Duval et al.^[^ ^28b^ ^]^ demonstrated this interference with formazan‐based viability measurements (MTT assay), suggesting that viability measurements for cellular biomineralization of GNPs should not rely on reporters with absorbance spectra overlapping with absorbance of GNPs.

Singh et al.^[^ ^28k^ ^]^ built on the work of Drescher et al.^[^ ^28i^ ^]^ in characterizing the effect of cellular biomineralization of GNPs on cellular viability with MCF7 human breast cancer cells. With the use of fluorescent calcein and propidium iodide staining, they found similar viability between 1.0 mm treatments of HAuCl_4_ and nontreated control MCF7 cells in full media. However, treatments made in SF media resulted in chromatin condensation indicating apoptosis.^[^ ^31^ ^]^ Following this analysis, authors characterized the senescent/quiescent state of cells by using *β*‐galactosidase (SA‐*β*‐gal) activity as a biomarker. These SA‐*β*‐gal measurements showed irreversible senescence in cells treated with concentrations of Au^3+^ greater than 0.50 mm in full media, even after application of a senescence‐reversal agent Y‐27632.^[^ ^32^ ^]^ Singh et al.^[^ ^28k^ ^]^ also compared absorption spectra and TEM of GNPs collected from the cell supernatant after treatments with Au^3+^ ions in full media and PBS. From these measurements, the authors found distinct differences in nanoparticle biomineralization between treatments in full cell media and PBS, with the treatment in full cell media resulting in spherical particles while the treatment in PBS resulting in sharp, faceted nanoparticles.

The data from Drescher^[^ ^28i^ ^]^ and Singh^[^ ^28k^ ^]^ indicated that gold biomineralization by cells under stress, whether through incubation in serum free(SF) PBS or with high concentrations of Au^3+^, results in differential nanoparticles compared with cells treated under normal growth conditions with a relatively low concentration of gold ions. Furthermore, fluorescent GNCs are preferentially formed by cells that are not stressed. This coincides with observations made in literature trends shown in Figure 4, wherein fluorescent GNCs are observed in treatments made in full cell media, and larger GNPs are found in treatments made in nutrient free conditions. Although this observation is insightful, it does not address the following important question: What are potential mechanisms driving cellular biomineralization of GNPs and GNCs?

## 5 Mechanism of Gold Biosynthesis

### 5.1 Reactive Oxygen and Nitrogen Species

There are many factors within cells that have the potential to enable GNP formation from ionic gold (Figure 2). Specifically, though, which of these factors and processes are actually involved, and how do they influence nanoparticle formation? Thus far, the majority of the existing work on this topic focuses on ROS/RNS as drivers of GNP biomineralization. One potentially important practical implication of ROS/RNS's involvement in this process is that they are upregulated in cancer pathology.^[^ ^28a,e–h,j^ ^]^ Contrary to the first publication by Anshup et al.,^[^ ^28a^ ^]^ a number of follow‐up studies presented evidence that nanoparticles are produced in greater extend by cancer cells than by noncancerous cells.^[^ ^28e–h,j–l^ ^]^ Wang et al.^[^ ^28e^ ^]^ showed that the formation of fluorescent GNCs through biomineralization in mammalian cells occurred more readily in cancerous human hepatoma (liver) HepG2 and human leukemia K562 than in “noncancerous” L02 cells treated with HAuCl_4_. It is important to note that the L02 cell line has since been found to be a HeLa derivative,^[^ ^33^ ^]^ calling into question its use as a noncancerous cell control; however, the oxidative stress elicited by H_2_O_2_ has been reported to be 25% lower for L02 cells than for HepG2 cells,^[^ ^34^ ^]^ so comparisons based on differences in ROS production can still be made.

In follow‐up studies by Dr. Wang's group, adjuvant treatments that affect cellular ROS/RNS formation were applied in order to affect cellular GNP/GNC formation.^[^ ^28g,j^ ^]^ The first study by Zhao et al.^[^ ^28g^ ^]^ investigated whether the co‐application of HAuCl_4_ and Fe^2+^ ions enhances formation of fluorescent GNCs as FeCl_2_ is known to induce an elevated ROS/RNS response.^[^ ^35^ ^]^ Indeed, the authors observed an enhanced fluorescence from GNCs associated with co‐application of FeCl_2_.^[^ ^28g^ ^]^ Later, Rehman et al.^[^ ^28j^ ^]^ included another adjuvant—sodium selenium (Na_2_SeO_3_), combined with FeCl_2_ and HAuCl_4_, that resulted in an additional increase in GNC fluorescence from treated cells. The rationale for this combination is that selenium is a known reducing agent for GNP formation^[^ ^36^ ^]^ and is a naturally occurring trace element as a component of selenocysteines/selenoproteins,^[^ ^37^ ^]^ so its application is not likely to be associated with any significant cytotoxicity.

### 5.2 NADH Dehydrogenase Flavoprotein 2 and Quinone Oxidoreductase‐Like Protein

**Figure 5:**
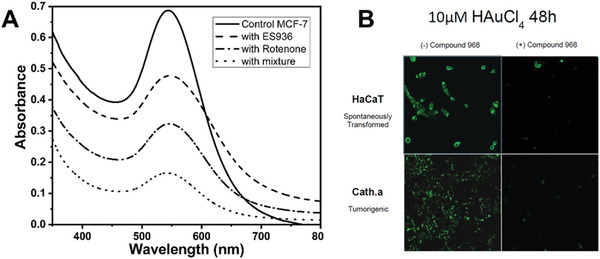
NADH dehydrogenase flavoprotein 2, quinone oxidoreductase‐like protein, and glutamate as drivers of gold nanoparticle biomineralization by mammalian cells. A) UV‐Vis spectra of gold nanoparticle solution that formed by mixing Au^3+^ with total protein of MCF‐7 cell lysate extracted from cells without added inhibitor (control) or treated with ES936, rotenone, and mixture of ES936 and rotenone inhibitors for 2 h (1, 2, 3, and 4), respectively. Reproduced with permission.^[^ ^28f^ ^]^ Copyright 2013, by WILEY‐VCH. B) Confocal micrographs of human keratinocytes (HaCaT) and mouse neuronal cells (Cath.a) treated with 10 µm HAuCl_4_ in full cell media for 48 h in either their native state or in the presence of the inhibitor Compound 968. Reproduced with permission.^[^ ^28h^ ^]^ Copyright 2016, by American Chemical Society.

Whereas the above‐mentioned studies indicated that ROS and RNS molecules can play a significant role in cellular biomineralization of gold through redox potential reactions, El‐Said et al.^[^ ^28f^ ^]^ were the first to directly explore the roles of specific biomolecules, namely, the role of NADH dehydrogenase (ubiquinine) flavoprotein 2 and of quinone oxidoreductase‐like protein (QOH‐1). Specifically, they used enzyme inhibitors rotenone and 5‐methoxy‐1,2‐dimethyl‐3‐[(4‐nitrophenoxy)methyl]‐1H‐indole‐4,7‐dione (ES936) to inhibit NADH and QOH‐1, respectively. In this inhibition study, 100 nm enzyme inhibitors were added to MCF‐7 breast cancer cells for 2 h; this treatment reduces the levels of these enzymes by more than 95% without impacting cell viability.^[^ ^38^ ^]^ Then the cells were lysed and the total protein of cells admixed with HAuCl_4_ for analysis of gold formation through measurements of absorption spectra. The inhibitions of NADH and QOH‐1 resulted in a significant decrease of nanoparticle plasmonic peak intensities (**Figure** **5**A), indicating potential role of these enzymes in cellular GNP biomineralization. However, this observation could partially be a consequence of the NADH binding capacity of these enzymes, as NADH is known to reduce nanoparticles spontaneously.^[^ ^39^ ^]^

### 5.3 Glutamate

In 2016, West et al.^[^ ^28h^ ^]^ further explored potential mechanisms of intracellular GNP formation through a different pathway, linking intracellular GNP reduction to glutamate synthesis.^[^ ^28h^ ^]^ They used a glutamate synthesis blocker compound 968 (Sigma, Saint Louis, MO) and found that in the presence of compound 968, fluorescence from intracellular GNCs was greatly reduced in noncancerous, spontaneously transformed, and tumorigenic cells (Figure 5B). These data suggest that glutamate may play a large role in intracellular formation of fluorescent GNCs and correlates with studies by Carera et al.,^[^ ^40^ ^]^ wherein GNPs were synthesized through direct reaction with sodium glutamate. However, glutamate is linked to many metabolic processes as well as being a precursor for a large variety of biomolecules,^[^ ^41^ ^]^ so it is possible that the observation by West et al.^[^ ^28h^ ^]^ could be associated with action of glutamate as a reducing agent or with downstream effects.

A better understanding of the mechanisms of gold particle biomineralization in mammalian cells could enable its potential use for biomedical applications. Appropriate pairing of its usage with diseases whose hallmarks are associated with the biological pathways involved in gold biomineralization would result in the greatest benefit. As discussed above initial work toward enabling this understanding showed some evidence that ROS,^[^ ^28e–h,j^ ^]^ NADH dehydrogenase flavoprotein 2,^[^ ^28f^ ^]^ quinone oxidoreductase‐like protein,^[^ ^28f^ ^]^ and glutamate^[^ ^28h^ ^]^ play a role in the formation of GNP biomineralization. However, it is likely that many biomolecules involved in this process, since Au^3+^ is a highly reactive gold state that can readily form nanoparticles upon interactions with a wide variety of biomolecules. Thus, explorations related to the influence of a single molecule may not be an efficient way of uncovering the mechanisms and pathways for cellular biomineralization of GNPs.

### 5.4 Protein Analysis

**Figure 6:**
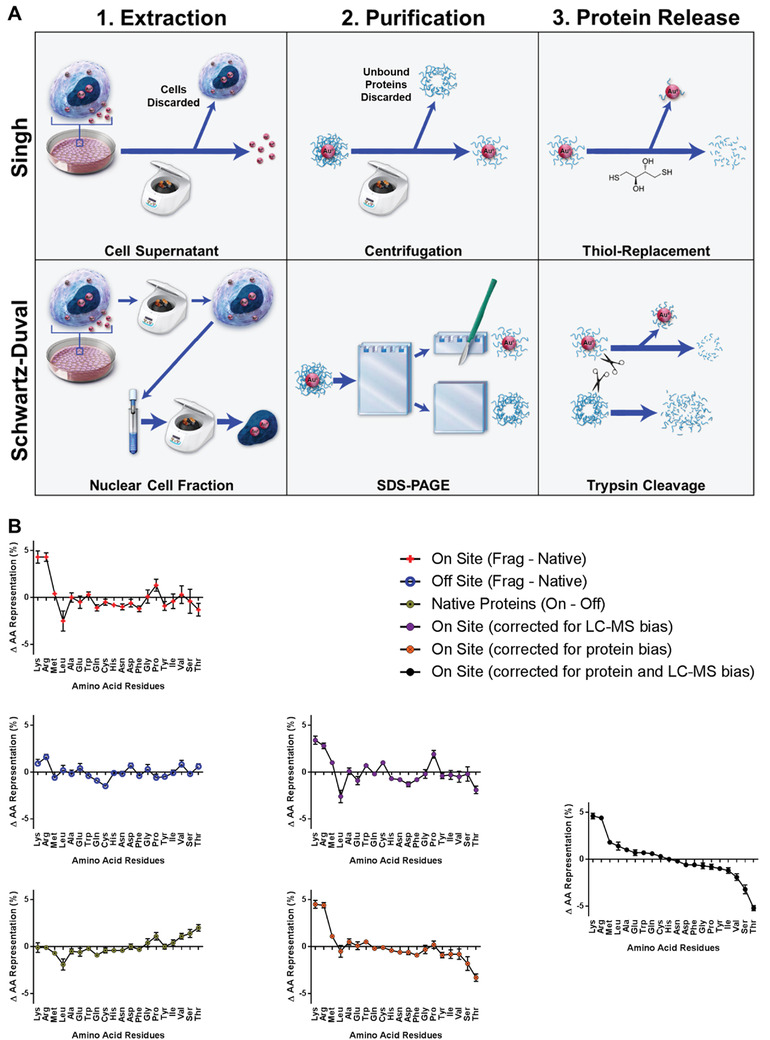
Preference in amino acid binding to in situ formed gold particles. A) Comparison of the processes used for 1) extraction of biomineralized GNPs, 2) purification of extracted biomineralized protein‐nanoparticle constructs from erroneous proteins, and 3) release of nanoparticle bound proteins preceding identification of proteins adhered to biomineralized GNPs through mass spectroscopy by Singh et al.^[^ ^28k^ ^]^ and Schwartz‐Duval et al.^[^ ^28b^ ^]^ B) Changes in amino acid (AA) distributions showing biases for AA distributions and bias corrected ranking of AA presence in identified protein fragments. AA distribution changes between on‐site proteins found in LC–MS fragments and native states in red. AA distribution changes between off‐site proteins found in LC–MS fragments and in native states in blue. AA distribution changes between complete sequences of on‐ or off‐site proteins in yellow. AA binding preferences with LC‐MS fragmentation bias correction in purple. AA binding preferences with bias correction between on‐ and off‐site proteins in orange. AA binding preference with bias correction for both protein and LC‐MS biases in black shows an overrepresentation of lysine and arginine and underrepresentation of serine and threonine. Error bars are standard deviations of the mean (*n* ≥ 16 proteins). Reproduced under the terms of a Creative Commons Attribution 4.0 International License.^[^ ^28b^ ^]^ Copyright 2020, Springer Nature.

Using high‐throughput methods, Singh et al.^[^ ^28k^ ^]^ and Schwartz‐Duval et al.^[^ ^28b^ ^]^ independently identified proteins that adhered to the GNPs/GNCs formed through biomineralization and the properties/characteristics of those proteins. Both studies used matrix‐assisted laser desorption ionization‐time of flight mass spectrometry (MALDI‐TOF MS) with MASCOT analysis (Matrix Science, Boston, MA) and MCF7 breast cancer cells for this work. However, there were distinct differences between the processes used for protein isolation and fragmentation preceding their respective analyses including 1) the subcellular location from which formed nanoparticles were extracted, 2) the separation of biomineralized nanoparticle‐protein constructs from erroneous proteins (i.e., proteins that were not associated with biomineralized particles), and 3) how the protein fragments were release from the extracted particles (**Figure** **6**A). Specifically, Singh et al.^[^ ^28k^ ^]^ collected nanoparticles from the supernatant of treated cells, whereas Schwartz‐Duval et al.^[^ ^28b^ ^]^ collected nanoparticles from the nuclear fraction of treated cells Additionally, Singh et al.^[^ ^28k^ ^]^ used a ligand exchange reaction with thiolated agents to free proteins adhered to GNPs through thiol bonds (Figure 6A), whereas Schwartz‐Duval et al.^[^ ^28b^ ^]^ performed the partial trypsin digestion of the total nanoparticle‐protein complexes before separating particles from partially digested protein fragments (Figure 6A). From the isolation techniques used by Singh et al., the authors were able to identify 10 different proteins that were chemically released from the particles (**Table** **1**). A number of these proteins have molecular functions related to DNA, ATP, and metal ion binding.^[^ ^42^ ^]^ Although the biomineralized nanoparticles used in this analysis were extracted from the supernatant space, surprisingly, only two of the nanoparticle‐bound proteins are typically found in the extracellular space—HSP70 and PUR9, and many are typically found within the nuclear compartment such as GTD2A, CPSF3, MT1X, and ZN681.^[^ ^43^ ^]^ Singh et al. then identified the specific gold‐binding peptide sequences among these proteins with use of a high‐throughput approach with custom peptide libraries (ranging from 7–13 mer length). The 7–13 mer peptides from the custom peptide library were incubated with gold ions for 24–72 h to determine which sequences were capable of reducing GNPs. Mixtures capable of reducing gold as well as specific binding kinetics were identified with use of a quartz crystal microbalance with dissipation monitoring (QCM‐D). Gold binding peptide sequences identified through this high‐throughput approach were attributed to specific identified proteins. Specific peptides rich in polar amino acid observed as good gold binders/reducers are shown in bold in Table 1.

**Table 1:** Proteins associated with extracellular gold particles produced through biomineralization. Major proteins, with gold‐binding peptide sequence, P‐value (frequency match would occur by chance), mass, molecular function, and number of polar amino acids (Polar AA) identified by Singh et al.^[^ ^28k^ ^]^ Polypeptides in bold show high affinity for gold‐binding/reducing as determined through phage display library results.^[^ ^28k^ ^]^

Gold binding proteins	Gold binding reducing polypeptide sequences	P‐value	Monoisotopic (Mr) (Mass in Daltons)	Molecular function	Polar AA
1‐*)ALG14‐UDP‐N‐acetylglucosamine transferase	VVAGSGGHTT NAYSPRHYVI	0.0041	24,135	Catalytic activity N‐acetyl glucose aminyl transferase activity	1,3
2‐ Cleavage and polyadenylation specificity factor subunit 3 (CPSF3)	FWCYHAGHVL	0.0059	73,000	Heavy metal binding	2
3 ‐Bifunctional purine biosynthesis protein (PUR9)	ANYWWLRHH	0.038	64,575	Cadherin binding involved in cell‐cell adhesion	4
4‐*)Heat shock protein (HSP70)	**YSENEEIVGLAAK**	0.011	54,760	ATP binding	2
5‐ Zinc finger protein (ZN681)	KAFNHSSHLATHK	0.033	75,011	DNA and metal ion binding	6
6‐ Archidonate 5‐lipoxygenase (LOX5)	**VMNHWQED** **YDWLLAK**	0.019	77,933	Iron ion binding	3,1
7‐ Serine/threonine‐protein kinase (KPSH1)	PENLLYYHPGTD	0.289	48,005	ATP binding protein Ser/Threo Kinase	2
8‐ Insulin receptor related protein (INSRRP)	**CWQPNPR**	0.055	14,3628	ATP binding actin cytoskeleton reorganization	3
9‐ General transcription factor II‐I (GTD2A)	TGTPAMVDANNG **IDEITDINNT**	0.009	107,162	DNA binding	2
10‐ Metallothionein‐1X	**GSYACAGSCKCK**	—	6,063	Metal binding	2

*) Proteins extracted from both serum/serum‐free culture of MCF7 incubated with gold ions.

From the isolation techniques used by Schwartz‐Duval et al.,^[^ ^28b^ ^]^ the authors identified 16 different proteins among fragments cleaved (via trypsinization) from the particles. Similar to findings by Singh et al.^[^ ^28k^ ^]^ these proteins have molecular functions related to DNA, ATP, antigen, and metal ion binding.^[^ ^42^ ^]^ Although the proteins from biomineralized nanoparticles were extracted from the nuclear fraction, surprisingly only three of these identified proteins are typically found within the nuclear compartment (TTN, FRG1, and DDX31).^[^ ^43^ ^]^ The method used by Schwartz‐Duval et al.^[^ ^28b^ ^]^ to release protein fragments from particles, by direct trypsinization cleavage of the whole particle‐protein complex, enables the identification of proteins that could be associated with particles through partial insertion or embedment within the particles. Furthermore, the use of direct trypsinization method ensures that the amino acids remaining bound to the gold surface will not appear in the LC‐MS MASCOT analysis; these amino acids are likely to play an important role in cellular biomineralization of gold. To identify these amino acids the authors ranked all amino acids based on their relative abundance within the identified fragments from free protein not adhered to particles and those excised from the gold constructs. To this end, amino acid distributions from identified fragments of on‐site proteins (i.e., proteins bound to biomineralized GNPs) and off‐site proteins (i.e., free protein not adhered to particles) were compared to normative amino acid distributions of the proteins in their native states (i.e., total amino acid sequence from UniProt library) (Figure 6B). Comparing amino acid distributions of protein fragments from erroneous proteins (off‐site) with the amino acid distribution of their whole unfragmented state (native state) enabled the authors to determine the bias of amino acids by the fragmentation process and LC‐MS analysis. Making this comparison of the amino acid distributions between native state gold‐bound proteins (on‐site) with erroneous non‐gold bound proteins (off‐site) enabled the authors to determine the bias of amino acid distribution between on‐site and off‐site proteins. After accounting for these biases, the authors determined that serine and threonine residues were the most underrepresented in the identified fragments of on‐site or gold‐bound proteins and thus these amino acids are most likely involved in gold intracellular biomineralization (Figure 6B). This coincides with previous reports ing interactions between specific polypeptides and gold indicating that close contact between peptides and gold (111) surfaces mainly involves polar side chains of serine and threonine.^[^ ^44^ ^]^ Surprisingly, there was no direct overlap between proteins identified by these two studies,^[^ ^28b,k^ ^]^ Although this is interesting and suggests that various proteins related to nuclear versus extracellular localization of biomineralized particles could exist, this lack of overlap could also be explained by the use of different protein extraction methods. However, there was overlap between identified protein functions. Specifically, many of the identified proteins in both studies were established as binding DNA or metal ions through searches of gene ontology database profiles.^[^ ^42^ ^]^ It is feasible that these identified proteins with cationic binding capacity (CPSF3, MT1X, ZN681, and PSKH1 from Singh et al.;^[^ ^28k^ ^]^ and GBE1, CDH23,CALM1, and TTN from Schwartz‐Duval et al.^[^ ^28b^ ^]^) may be directly involved in the biomineralization of gold in MCF7 cells initiated through a gold ion replacement. There is considerable evidence of gold‐ion replacement within metalloproteins,^[^ ^45^ ^]^ already with more than 80 known structures of gold metallation proteins.^[^ ^45c^ ^]^ Endogenous biomolecules such as metalloenzymes with cationic binding sites (such as Fe, Ca, Zn, Cu) are known to have promiscuity in their cationic binding.^[^ ^45b^ ^]^ While the catalytic activity of these molecules may be reduced, there is some evidence that partial bioactivity can be retained, with examples of this observed through Co^2+^ or Cd^2+^ replacement.^[^ ^45d–f^ ^]^ Additional support of the potential role of metalloproteins in cellular biomineralization of gold comes from the findings within the report by Balfourier et al.^[^ ^27b^ ^]^ concerning the long‐term dissolution and recrystallization of prefabricated GNPs—particularly the mechanisms of recrystallization. They showed that GNPs can be degraded via ROS generated species within the lysosomes and, then recrystallized via metallothioneins. Interestingly, there is little overlap between the specific biomolecules involved in gold biomineralization identified in reports by Balfourier et al.,^[^ ^27b^ ^]^ Singh et al.,^[^ ^28k^ ^]^ and Schwartz‐Duval et al.^[^ ^28b^ ^]^ with only metallothione‐1X (MT1X) being identified by both the Balfourier et al.^[^ ^27b^ ^]^ and Singh et al.^[^ ^28k^ ^]^ The lack of overlapping biomolecules could reflect differences in composition and environment of intracellular compartments that are involved in gold biomineralization. Indeed, among the proteins identified in the reports from Singh et al.^[^ ^28k^ ^]^ and Schwartz‐Duval et al.^[^ ^28b^ ^]^ a majority are nuclear proteins (TTN, FRG1, DDX31, GTD2A, ALOX5, CPSF3, MT1X, ZN681, PSKH1, HSP70), however, some are from other subcellular regions such as the cell membrane (IGHA1, CD23, PSKH1, PUR9), cytosol (GBE1, ALOX5, HSP70), cytoskeleton (CALM1, PSKH1, HSP70), endoplasmic reticulum (PSKH1), and some are from the extracellular space as secretions (GBE1, IGLL5, IGHA1, HSP70, PUR9) or as components of exosomes (GBE1, HSP70, and PUR9).^[^ ^43^ ^]^ The report by Balfourier et al.,^[^ ^27b^ ^]^ is focused on lysosomes—the subcellular location where they had identified gold particle dissolution and recrystallization to occur; this is distinctly different from the reports by Singh et al.^[^ ^28k^ ^]^ and Schwartz‐Duval et al.^[^ ^28b^ ^]^ where whole cells were treated with gold ions to initiate the biomineralization process. Interestingly, these differences in environmental conditions of various cellular compartments result in strikingly different morphology of biomineralized GNPs (compare morphologies shown in Figures 1I and 3A).

## 6 Loci of Cellular Particle Biosynthesis

Understanding the subcellular locations for GNP biomineralization and the respective mechanisms involved therein, would better inform applications of use similar to how understanding the therapeutic mechanisms of chemodrugs enables their targeted use.^[^ ^46^ ^]^ Reports of gold biomineralization by mammalian cells identified gold particles in the extracellular space^[^ ^28a,f,j–l^ ^]^ as well as intracellularly.^[^ ^28b–e,g–j^ ^]^ However, it is not clear if particles are formed in these observed locations or migrate there after biomineralization. Since currently there is a limited number of studies in mammalian cells, additional mechanistic information could be gained from studies of microbial gold biomineralization where three loci have been considered in nanoparticle nucleation: 1) extracellularly via secreted metallophore biomolecules, as a stress response to remediate ion toxicity;^[^ ^17t^ ^]^ 2) the outer cell membrane or cell wall, wherein ions passively accumulate followed by nucleation and precipitation of nanoparticles or other larger nanostructures;^[^ ^17s^ ^]^ and 3) intracellularly, where gold ions are reduced to particles, to reduce cytotoxicity.^[^ ^17o^ ^]^ It was shown that the ensuing gold particles produced via microbial biosynthesis are distinct by loci of formation, with particles inside microbes being considerably smaller (<10 nm) than those reduced externally either by secretions or on the outer membrane (1–10 µm).^[^ ^17j,ab–ae^ ^]^ Thus far, no reports on mammalian gold particle biosynthesis meaningfully compare sizes of gold nanostructures depending on their formation loci. In this section we focus on biomineralization of GNPs in extracellular, intracellular, and the boundary (i.e., cell membrane) loci of prokaryotic and eukaryotic cells.

Secreted proteins related to gold biomineralzation by mammalian cells include proteins that are related to stress responses such as antigen binding (IGLL5, and IGHA1) and protein unfolding (HSP70).^[^ ^28^, ^42^ ^]^ This finding correlates with microbial GNP synthesis, showing secretion of metallophore moieties (such as delftibactin) as a response to stress that results in precipitation of ionic gold to a stable particle form.^[^ ^17t,z^ ^]^ The time‐frame of particle synthesis via microbial secretions is known to occur within 30 min from the start of the ion treatment.^[^ ^17t^ ^]^ Within mammalian systems, the shortest time‐point recording extracellular nanoparticle formation was 24 h, with treatments in PBS by El‐said et al.,^[^ ^28f^ ^]^ and Anshup et al.,^[^ ^28a^ ^]^ as well as both serum and serum free media conditions by Singh et al.^[^ ^28k^ ^]^ There may be particle formation at earlier time points, however, these measurements were not carried out.

Membrane mediated nanoparticle synthesis was directly observed by Singh et al.^[^ ^28k,l^ ^]^ and by Schwartz‐Duval et al.^[^ ^28b^ ^]^ Specifically, Singh et al.^[^ ^28k,l^ ^]^ showed gold nanostructures covereing the cell's surface using electron microscopy; and Schwartz‐Duval et al.^[^ ^28b^ ^]^ observed plasmonically enhanced Raman signal in the membrane of intact cells and in membrane fractions of lysed cells pre‐treated with gold ions. However, there is also, potential, indirect evidence of particle nucleation on the cell membrane, through the report indicating the presence of biosynthesized gold particles in exosomes (Figure 8A,B),^[^ ^28j^ ^]^ as exosomal vesicles are derived from the cell membrane. This membrane‐mediated synthesis was thoroughly explored in microbial systems by Kang et al.^[^ ^17s^ ^]^ In this study, they showed that extracellular polymetric substances (EPS), such as polysaccharides and proteins, are largely responsible for membrane‐associated synthesis by showing that removal of EPS reduced GNP formation on the membrane. Further, validating this finding, they showed that extracted EPS moieties can directly reduce Au^3+^ to GNPs in a concentration dependent manner. A similar observation was made in mammalian systems where freshly extracted cell membranes from B16‐F10 mouse melanoma cells were shown to spontaneously reduce Au^3+^ to gold particles;^[^ ^39a^ ^]^ although the specific molecular mechanism was not addressed in this study, the suggested role of polysaccharides and proteins from bacterial evaluation by Kang et al.^[^ ^17s^ ^]^ provides a viable hypothesis for future mechanistic exploration of mammalian systems.

The intracellular nanoparticle formation by mammalian cells is evident at the earliest measurement points of ≈30–60 min by higher Raman and fluorescence signals associated with SERS and GNCs, respectively, from treatment groups compared against non‐treated controls.^[^ ^28b,g^ ^]^ For example, Schwartz‐Duval et al.^[^ ^28b^ ^]^ used Raman microscopy to confirm formation of plasmonic gold particle following vectorized delivery of Au^3+^ as early as 30 min after treatment with a continued increase in Raman signal at 1, 2, and 4 h after treatment indicating continuing GNP formation. Zhao et al.^[^ ^28g^ ^]^ explored fluorescent properties of GNCs to evaluate timing of their formation in cell lines (HepG2 and L02) and in subcutaneous xenograft mouse models with U87 and HepG2 human cancer cells. In vitro, they found an increase in fluorescence signal due to intracellularly formed fluorescent GNCs as early as 1 h post‐gold ion treatment with a continuous increase of fluorescence up until ≈7 h where the signal intensity plateaued for Au^3+^ treatment alone and in combination with Fe^2+^ that was co‐applied to increase gold biomineralization. In vivo, they observed that fluorescence for their combined treatment peaked at ≈20 h, whereas the fluorescence intensity following Au^3+^ treatment alone did not peak even at the latest time point of 24 h. Similarly, the study by Schwartz‐Duval et al.^[^ ^28b^ ^]^ reported an increase in the fluorescent signal at the site of injection of polymer‐encapsulated Au^3+^ ions peaking at 48 h after injection and returning to background signal after 7 days (Figure 9F).

Considering the internalization of ionic gold precursor material, cellular uptake could occur through “borrowed” pathways used by other ions,^[^ ^47^ ^]^ common nanoparticle endocytotic pathways,^[^ ^48^ ^]^ or a combination of both. The report from Schwartz‐Duval et al.^[^ ^28b^ ^]^ explored nanoparticle endocytosis pathways via microarray and blocking studies (quantifying gold content via ICP‐MS), finding that there were very few changes in gene regulation related to endocytosis and that none of the inhibitors indicated preferential uptake. Similarly, the report by Drescher et al.,^[^ ^28i^ ^]^ found that cells treated with prefabricated particles had considerably lower gold content compared to either condition when treated with ions. These data suggest that gold ion internalization occurs through pathways distinct from nanoparticle internalization.

Currently available reports of gold biomineralization by mammalian cells identified a number of important parameters of this process including kinetics of formation of gold nanostructures, sizes, and physical properties of biosynthesized GNPs, their cellular localization as well as provided initial mechanistic insights into the biomineralization process. This knowledge has already been transferred into initial biomedical applications that we discuss below.

## 7 Biomedical Potential of Cells as Nanoparticle Factories and In Situ Nanomedicine

**Figure 7:**
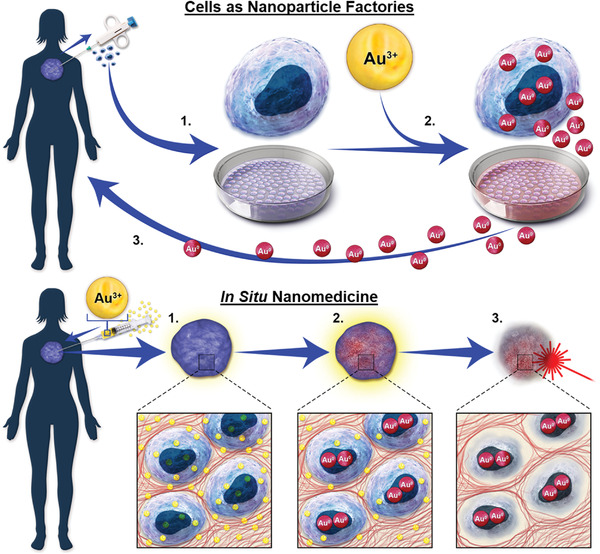
The ideological duality for the application of cellular biomineralization of GNPs. *Cells as Nanoparticle Factories*, wherein patient's cells are 1) cultured from biopsy and 2) used as bioreactors to create personalized biomimetic nanoparticles in order to 3) reapply to the patient; and in situ *Nanomedicine*, wherein 1) ionic gold is applied directly to the pathological tissue, 2) resulting in the formation of nanoparticles within that tissue 3), enabling imaging or a therapeutic treatment such as fluorescence or photothermal ablation respectively.

The studies of gold biomineralization in mammalian cells reveals an ideological duality related to the potential application of this process by using cells as biomimetic nanoparticle factories, and through in situ nanomedicine (**Figure** **7**). Cells as biomimetic nanoparticle factories were presented by Anshup,^[^ ^28a^ ^]^ El‐Said,^[^ ^28f^ ^]^ Rehman,^[^ ^28j^ ^]^ and Singh,^[^ ^28k^ ^]^ who used cells as incubators to form biomimetic GNPs to be applied or envisioned to be applied elsewhere. In situ nanomedicine has been presented by Shamsaie,^[^ ^28c^ ^]^ Liu,^[^ ^28d^ ^]^ Wang,^[^ ^28e^ ^]^ Zhao,^[^ ^28g^ ^]^ West,^[^ ^28h^ ^]^ Drescher,^[^ ^28i^ ^]^ Rehman,^[^ ^28j^ ^]^ Singh,^[^ ^28l^ ^]^ and Schwartz‐Duval,^[^ ^28b^ ^]^ who described cells forming GNPs as the potential target, relying on the differential nanoparticle formation for diagnosis or therapeutic applications.

### 7.1 Cells as Biomimetic Nanoparticle Factories

In 2005, Anshup et al.^[^ ^28a^ ^]^ were the first to publish the concept of giving cells raw materials to produce biomimetic gold particles. El‐Said et al.^[^ ^28f^ ^]^ showed that through variations in incubation time and Au^3+^ concentration, a wide variety of GNPs can be formed.

**Figure 8:**
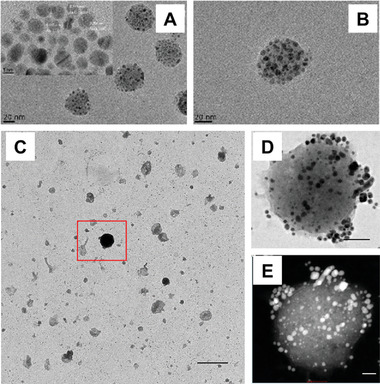
Comparison of gold particles loaded within exosomes through cellular biomineralization and manual benchtop preparation. Gold nanoparticle containing exosomes formed A,B) through cellular reduction of HAuCl_4_ with the addition of iron (II) chloride to promote cellular production of ROS/RNS and C–E) through benchtop synthesis via mixing of exosomes with HAuCl_4_. A,B) Reproduced with permission.^[^ ^28^ ^]^ Copyright 2018, Elsevier. C‐E) Reproduced with permission.^[^ ^49^ ^]^ Copyright 2019, Elsevier.

Next, Singh et al.^[^ ^28k^ ^]^ characterized the diverse nature of proteins functionalizing GNPs that are formed this way. In addition, Rehman et al.^[^ ^28j^ ^]^ showed that nanoparticles formed through biomineralization are present within extracellular vesicles generated by cells pretreated with gold salts (**Figure** **8**A,B). The authors concluded that GNPs were formed intracellularly and then loaded within exosomes. However, there is also a possibility that the GNPs could be reduced on or by exosomes extracellularly, which was reported elsewhere (Figure 8C–E).^[^ ^49^ ^]^ To assess whether the GNPs found in the extracellular space are produced mostly by gold reduction on extracellular vesicles or if they are first formed inside cells followed by cell excretion in extracellular vesicles would be an important experiment for potential biomedical applications. Using cells as biomimetic nanoparticle factories may have significant potential, especially if the nanoparticles formed this way are packaged within the cell‐excreted exosomes, as exosomes are involved in cell‐cell communication and in turn the tumor niche formation.^[^ ^50^ ^]^ If nanoparticles produced by cells are packaged within exosomes, then there is a possibility for exploiting this exosomal communication system for distribution of therapeutic particles within the tumor niche and possibly even to metastatic sites. While not exploring this exosomal delivery directly, two of the particle‐bound proteins identified by Singh et al.,^[^ ^28k^ ^]^ namely, HSP70 and PUR9 are proteins found on exosomes. This study also explored homotypic targeting and uptake of extracellular gold particles produced by MCF7 and C2C12 cells.^[^ ^28k^ ^]^ It was shown that both cells preferentially uptake homotypic nanoparticles with MCF7 cells showing an ≈ 2.5‐ and ≈5.0‐fold homotypic particle uptake preference over particles synthetized by C2C12 cells and chemically synthetized nanoparticles, respectively, and C2C12 cells exhibiting a ≈3.3‐ and approximately ≈5‐fold homotypic uptake preference over nanoparticles produced by MCF‐7 biomineralization and chemical synthesis, respectively.^[^ ^28k^ ^]^ This homotypic targeting supports the notion of exosomal packaging, as there is prior evidence that exosomes from cancer cells can act as a “trojan‐horse” to deliver cancer drugs.^[^ ^50c^ ^]^ Building from this, if the pathological tissue itself is used as a nanoparticle factory, then only gold salts would need to be administered to the tissue, and as discussed above, gold salts have a lengthy tenure of clinical use. We refer to this strategy, wherein gold salts are applied directly to the pathological tissue, resulting in the formation of biomimetic GNPs from that tissue for the purpose of therapy or diagnosis against that pathology, as “in situ nanomedicine.” Under this paradigm, theranostic nanoparticles could be generated in situ within the tissue, and the theranostic properties would be dependent on the tissue properties.

### 7.2 In Situ Nanoparticle Synthesis for Diagnosis and Therapy

#### 7.2.1 In Situ Synthesis of Particles to Provide Imaging Contrast

The 2007 paper by Shamsaie et al.^[^ ^28c^ ^]^ was the first to present the strategy of generating GNPs intracellularly as an alternative to cell targeting using prefabricated nanoparticles. Using the same incubation parameters as Anshup et al.^[^ ^28a^ ^]^ but with a different cell line, normal human breast epithelial MCF10a, the authors presented their approach as a solution to the challenges of endosomal escape and nuclear delivery of GNPs as Raman probes. GNPs that are suitable for SERS are too big for efficient nuclear delivery without developing a complex nanoparticle design and they typically aggregate in endosomes.^[^ ^51^ ^]^ Growth of GNPs as Raman reporters intracellularly was shown as a valid strategy resulting in SERS signals from the nucleus and the cytosol. Single gold ions are considerably smaller than nanoparticles and, therefore, can easily penetrate cellular organelles such as the nucleus where in situ gold biomineralization can result in formation of GNPs that provide a strong SERS signal. Shamsaie et al.^[^ ^28c^ ^]^ compared Raman spectra of cells with intracellularly grown GNPs and cells incubated with prefabricated 10 and 50 nm diameter GNPs. They observed a striking difference between the Raman spectra from the intracellularly grown particles compared against prefabricated particles, especially, at the 500 cm^–1^ band. This band that is associated with the stretching vibration mode of the disulfide bond (v S‐S),^[^ ^52^ ^]^ is favorably formed and observed within the oxidizing environment of lysosomes.^[^ ^53^ ^]^ It was observed for prefabricated particles in the Shamsaie et al. and other reports^[^ ^28^, ^54^ ^]^ and it was absent in the case of intracellularly formed particles. This data indicates that pre‐fabricated GNPs might be trafficked to lysosomes following cellular internalization, while intracellularly grown nanoparticles can localize in various cellular compartments as discussed above. Subsequently, Liu et al.^[^ ^28d^ ^]^ were the first to explore how adjuvant treatments such as graphene oxide could accelerate the intracellular formation of GNPs as intracellular Raman probes in human lung cancer (A549), mouse breast cancer (4T1), and human cervical cancer (HeLa) cells. They found that the addition of graphene oxide could increase the intracellular SERS signal and could reduce the time required to form intracellular Raman reporter probes to ≈15 h compared with ≈24 h treatments required in the case of Au^3+^ alone. This study showed that the majority of intracellularly formed GNPs localized to the nucleus with or without the use of graphene oxide adjuvant; this observation of nuclear localization was similar to the two previous reports by Anshup^[^ ^28a^ ^]^ and Shamsaie.^[^ ^28c^ ^]^ Most recently, Schwartz‐Duval et al.^[^ ^28b^ ^]^ were able to reduce the incubation time even further through the nano‐vectored application of Au^3+^ combined with polyethylene glycol (PEG) by observing SERS signal in the treated MCF7 cells in vitro as early as 30 min after application of Au^3+^, with the maximal Raman signal achieved within 4 h (Figure 3E).

**Figure 9:**
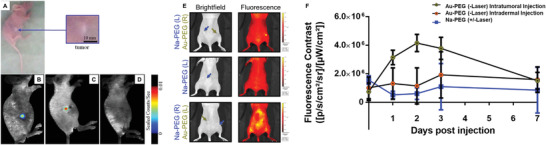
In situ nanomedicine for fluorescent imaging contrast. A photo of a mouse with A) a subcutaneous hepatocellular carcinoma and B) in vivo fluorescence image of intratumorally generated fluorescent GNCs, 24 h after a subcutaneous injection of 10 mm HAuCl_4_. C) In vivo fluorescence image of chronic myeloid leukemia 24 h after 10 mm injection of HAuCl_4_ near the tumor. D) In vivo fluorescence image of a control (non‐tumor bearing) mouse 48 h after 10 mm injection of HAuCl_4_ in the right side of the abdomen. E) Bright field and in vivo fluorescence images xenograft tumors treated either with mixtures of 7.5 mm Au^3+^ with PEG (Au‐PEG) or the same molar concentration of sodium chloride PEG mixture (Na‐PEG, control); the top and the middle images show intratumoral injections and the bottom images are from a transdermal injection (administered locoregionally to the tumor). F) Longitudinal assessment of fluorescence contrast comparing intratumoral and intradermal injections of ionic gold (Au‐PEG) and salt control (Na‐PEG) in mice that were used in photothermal therapy study (“+/‐“ indicates whether a laser was applied during treatment). A–D) Reproduced under the terms of a Creative Commons Attribution 4.0 International License.^[^ ^28e^ ^]^ Copyright 2013, Springer Nature. E,F) Reproduced under the terms of a Creative Commons Attribution 4.0 International License.^[^ ^28b^ ^]^ Copyright 2020, Springer Nature.

In addition to providing Raman enhancement through plasmonic nanoparticle formation, fluorescent GNCs may also form through cellular biomineralization of Au^3+^.^[^ ^28b,e,g,h,j^ ^]^ These particles are commonly imaged by using 450–490/540–840 nm excitation/emission filter pairing.^[^ ^28b,e,g,h,j^ ^]^ Among these studies, the majority of in vivo animal applications focused on fluorescent nanoparticle formation within tumors. Wang's group^[^ ^28e^ ^]^ presented data suggesting that subcutaneous tumor xenografts have a higher capacity for in situ formation of fluorescent GNPs through biomineralization and that even injections of gold salts near the tumor can provide fluorescent contrast (**Figure** **9**B–D). However, the latter observation was not supported by the recently published work by Schwartz‐Duval et al.,^[^ ^28b^ ^]^ where fluorescence GNC formation was observed throughout the subcutaneous space if not injected directly into the tumor (Figure 9E,F).

#### 7.2.2 In Situ Therapeutic Particles

**Figure 10:**
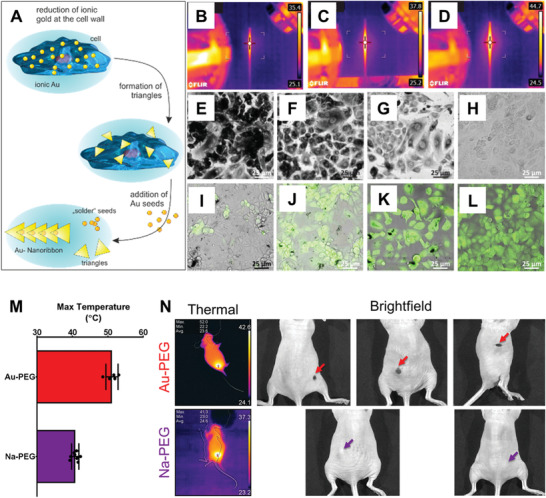
In situ nanomedicine for photothermal therapy. Photothermal therapy applications of cellular biomineralization of ionic gold by Singh et al.^[^ ^28l^ ^]^ and Schwartz‐Duval et al.^[^ ^28b^ ^]^ A) Schematic of a multistep synthesis of Au nanoribbons by action of cellular biomineralization of ionic gold combined with an application of gold seeds.^[^ ^28l^ ^]^ Thermograms of microcapillaries filled with B) spherical gold NPs, C) PEGylated gold nanorods, and D) nanoribbons under laser irradiation. Optical images of cells after laser irradiation stained with either E–H) trypan blue or I–L) calcein AM; prior to laser irradiation the cells were subjected to the following treatments: E,I) intracellular generation of nanoribbons, F,J) PEGylated‐Au nanorod, G,K) spherical gold NPs, and H,L) control cells without any treatment.^[^ ^28l^ ^]^ M) Maximum surface temperature from laser irradiation to tumors treated with either vectorized ionic gold (Au‐PEG, red) or a sodium chloride control (Na‐PEG, purple) control via thermal imaging.^[^ ^28b^ ^]^ N) Thermal and bright field images from laser treatment of M with colored arrows indicating either remaining tumor from Na‐PEG treatments (purple) or cavities/scabbing from total tumor elimination via photothermal effect through Au‐PEG (red) treatments.^[^ ^28b^ ^]^ A‐L) Reproduced under the terms of the Creative Commons CC‐BY license.^[^ ^28l^ ^]^ Copyright 2018, Royal Society of Chemistry. M,N) Reproduced under the terms of a Creative Commons Attribution 4.0 International License.^[^ ^28b^ ^]^ Copyright 2020, Springer Nature.

Additional efforts were focused on exploring the therapeutic potential of GNPs formed through cellular biomineralization in photothermal ablation.^[^ ^28b,l^ ^]^ To this end, Singh et al.^[^ ^28l^ ^]^ explored treatments combining cellular biomineralization of ionic gold with prefabricated nanoparticles (**Figure** **10**A).^[^ ^28l^ ^]^ In this approach, HAuCl4 in PBS was first directly added to confluent human breast adenocarcinoma MCF7 cells in full cell media, and after 8 h of incubation, was followed by addition of 20‐nm spherical GNPs with an additional 24–36 h incubation (Figure 10A).^[^ ^28l^ ^]^ This process induced growth of anisotropic gold nanostructures (nanoribbons) that were better suited for a photothermal treatment than prefabricated PEGylated gold nanorods. This conclusion was supported by thermal imaging of photothermal treatments (Figure 10B–D), as well as by evaluation the efficacy of photothermal treatment in killing cancer cells (Figure 10E–L). From thermal imaging of 780 nm NIR laser excitation of particles in DMEM at ≈0.6 W cm^−2^, the nanoribbons had a photothermal effect 15.3 °C greater than the nanorods and 26.4 °C greater than the spherical particles (Figure 10B–D). After this in vitro study, Schwartz‐Duval et al.^[^ ^28b^ ^]^ used a vectorized application of ionic gold for plasmonic nanoparticle generation in vivo (60 µg Au per mouse). The authors used a 10 kDa hydroxyl terminated PEG as a delivery vehicle for the ionic gold in direct intratumoral injections in MCF7 mouse xenografts.^[^ ^28b^ ^]^ In this approach, the authors relied on the natural tendency for PEG to form clusters under acidic conditions. Thus, the presence of HAuCl_4_ resulted in a coaggregation of PEG with the Au^3+^ ions in ≈200 nm clusters that prevented a prematurely reduction of gold ions before interacting with cells. The study by Schwartz‐Duval et al.^[^ ^28b^ ^]^ marks the first photothermal therapeutic treatment in vivo by GNPs generated within the tumor (Figure 10M,N). Tumors treated with the Au^3+^‐PEG clusters were completely eliminated after only two 3 min of exposures to a 500 mW 632 nm CW laser increasing tumor temperatures by 10.4 °C greater than controls (Figure 10M,N).^[^ ^28b^ ^]^ Currently there are no studies directly comparing in situ nanoparticle generation with treatments using prefabricated GNPs. However, some initial comparisons could be made using available data on treatment conditions. For example, Mulens‐Arias et al.,^[^ ^8f,g^ ^]^ reported a therapeutic photothermal effect in vivo with prefabricated particles using 1/3 of the total gold content used by Schwartz‐Duval's in situ synthesis,^[^ ^28b^ ^]^ but using a laser with four times greater power (2 W cm^−2^). Similarly, the preeminent studies describing enhancement of photothermal therapy using pre‐fabricated GNPs such as the 2003 report by Hirsch et al.,^[^ ^8c^ ^]^ which used gold nanoshells, and the subsequent 2019 clinical study by Rastinehad et al.,^[^ ^13c^ ^]^ applied 35 W cm^−2^ and 4.5–6.5 W cm^−2^ treatments, respectively, that is significantly higher power than used in studies exploring in situ synthesis −0.5 and 0.6 W cm^−2^.^[^ ^28b,l^ ^]^

## 8 Future Outlooks

From these reports, we can begin to see the potential for cellular biomineralization of GNPs as an alternative to the classic benchtop preparation, perhaps using cells as biomimetic nanoparticle factories, in situ nanomedicine, or in a combination of both. The published studies have demonstrated these processes providing SERS and fluorescence image contrast as well as photothermal therapy.

This emerging approach can have significant clinical potential because it is associated with an ultimate size reduction in delivery of a gold therapeutic agent to just a single atom that could greatly improve transport within dense biological environments. Moreover, gold salts have decades‐long history of a safe clinical use in treatment of rheumatoid arthritis providing a clear path toward clinical translation. Through cellular biomineralization of gold particles from salts, this pathway toward clinical translation can be combined with the vast wealth of peer reviewed published knowledge relating to GNPs. Additionally, this application is inherently simplistic, with a single active component—gold ions, but it takes advantage of a complex cell biology in order to produce therapeutic GNPs inside mammalian cells. However, this strategy also has its own unique challenges including a number of critical questions that need to be addressed to facilitate clinical translation: 1) is the time‐frame for biosynthesis adequate for clinical applications? 2) What are the transport phenomena of biomineralization precursor materials and byproducts? 3) What is the location of therapeutic action? 4) How can the toxicity of soluble heavy metals be addressed? 5) Are there any ways to control biosynthesis for desirable outcomes? Below we summarize initial considerations based on the available literature reports.

### 8.1 Is the Time‐Frame of Cellular Biosynthesis Appropriate for Clinical Use?

From studies covered herein, we can see that the initiation of nanoparticle formation occurs fairly quickly, within 30 min following administration of gold ions, as was assessed by fluorescence imaging and Raman spectroscopy with the maximum nanoparticle formation intracellularly occurring within 24–48 h.^[^ ^28b,g^ ^]^ Comparatively, the dissolution of GNPs is on the scale of weeks to months.^[^ ^27b^ ^]^ One could imagine applications of gold ions for cellular biomineralization through staging similar to the clinical study conducted using gold nanoshells for photothermal ablation of prostate cancer.^[^ ^13c^ ^]^ The clinical study was staged as a 2‐day process, wherein patients received nanoparticle infusion 1 day before the photothermal therapy. Similarly, with gold biomineralization, ions could be applied 1–2 days prior to therapy or diagnosis.

### 8.2 What Transport Phenomena Are Involved?

Understanding transport phenomena involved in mammalian gold particle biomineralization will also be essential for successful clinical translation. It will be important to understand how much gold will be retained at the delivery site following local administration or how effectively gold ions can be delivered through systemic route (e.g., by potential utilization of delivery vehicles discussed below in Section 8.5.2). Since gold ions can be easily reduced by a variety of biologically abundant molecules,^[^ ^55^ ^]^ it is likely that following local administration most of the delivered dose will be reduced to particles locally. This hypothesis is supported by a high local fluorescence of GNCs observed in vivo by both Zhao et al.^[^ ^28g^ ^]^ and Schwartz‐Duval et al.^[^ ^28b^ ^]^ after intratumoral injection of gold ions (Figures 9). Therefore, the relevant transport phenomena for gold ions is likely to be primarily limited to the local environment per application and the systemic transport would primarily be associated with biomineralized nanoparticles. Once formed, the fate of in situ formed GNPs would likely be comparable to other particles of similar size reported in literature.^[^ ^56^ ^]^ Size dependent trends within these reports indicate that larger particles (>10 nm) tend toward a biliary excretion through the fecal route and smaller particles (< 10 nm) excreted via urinary route. However, the surface coating also has significant influence on nanoparticle biodistribution,^[^ ^4^ ^]^ especially when considering specific targeting.^[^ ^4d,e,g–i^ ^]^ As discussed herein, the size and surface coating of biomineralized GNPs produced by mammalian cells are dependent on the cellular microenvironment in which they are formed, although it remains to be determined if the particle bound biomolecules retain their activity. Overall, the biodistribution of biosynthesized GNPs is determined by local diffusion of gold ions with a concurrent reduction process in the extracellular space and various cellular compartments that can also involve potential diffusion and transport of the formed nanoparticles. Understanding these transport phenomena will in turn identify potential loci for therapeutic action of biomineralized nanoparticles.

### 8.3 What are Potential Locations of Therapeutic Action?

Treatments with gold ions for nanoparticle biomineralization present many potential locations for therapeutic action through distinctions between each nucleation loci (e.g., extracellularly, on the membrane, and intracellularly). For instance, if a disruption of the cell membrane is desired, then strategies to enhance membrane templated synthesis could be applied through targeting membrane moieties (as discussed below in Section 8.5.1.). Further, extracellular particles loaded in exosomes have the potential to target distal pathologies. However, we find the innate property of nuclear accumulation to be of particular interest, as the nucleus of cells and the nucleic acids therein are highly desirable targets for therapy.^[^ ^8h^ ^]^ Multiple studies have been focused on development of complex strategies to accomplish delivery to this cellular compartment.^[^ ^4g–i^ ^]^ For example, Pan et al.^[^ ^8h^ ^]^ showed that nuclear delivery of pre‐synthetized gold nanorods can greatly enhance efficiency photothermal therapy of cancer cells in vivo with only a mild photothermal effect using a low intensity laser of 0.2 W cm^−2^. Further, an efficient photothermal therapeutic treatment of cancer in vivo with in situ synthetized GNPs reported by Schwartz‐Duval et al.^[^ ^28b^ ^]^ required ≈70‐fold lower power than was used in the foundational study by Hirsch et al.^[^ ^8c^ ^]^ that employed pre‐synthetized gold nanoshells without nuclear targeting that ultimately lead to clinical trials.^[^ ^13c^ ^]^ Notwithstanding that these are separate studies, the apparent difference in efficiency of photothermal therapy might be associated with nuclear localization of biomineralized GNPs in the study by Schwartz‐Duval et al.^[^ ^28b^ ^]^ However, while the nucleus is a desirable target for some therapeutic interventions, there is also evidence showing that nuclear localization of gold particles may induce cytotoxicity.^[^ ^57^ ^]^ Further, nanoparticle biomineralization that involves nuclear binding proteins might disrupt their function and, therefore, lead to unanticipated adverse effects. These potential undesirable consequences of gold biomineralization process will need to be carefully studied especially in normal cells.

### 8.4 Addressing the Toxicity of Soluble Heavy Metals

With treatments of gold salts, intended to generate nanoparticles within the affected cells, there may be concerns regarding safety especially considering the known cytotoxicity of HAuCl_4_ and other soluble heavy metals. While HAuCl_4_ treatments can be associated with cytotoxicity in some cells with doses as low as 0.25 mm,^[^ ^28i^ ^]^ the biomineralized GNPs are stable and are considered bioinert with low cytotoxicity as has been well‐established in both biomedical and geobiology research communities.^[^ ^6^, ^17^ ^]^ Compared with the rapid reaction kinetics for gold particle reduction from ions, the dissolution kinetics of gold (conversion of Au^0^ to Au^3+^ or Au^1+^) through microbial biomineralization is so slow that episodes of gold cycling (dissolution and reprecipitation) are estimated to occur on the time scale of years (≈7.64–4.1 years per cycle).^[^ ^17r^ ^]^ This slow, rate‐limiting dissolution is in‐part what enables the low cytotoxicity of gold particles.^[^ ^17p,r,s^ ^]^ In microbial settings, mineralization of metal ions serves as a mechanism to reduce toxicity^[^ ^17n^ ^]^ that can potentially be maintained continuously as long as cell metabolism remains intact.^[^ ^17l–n,y^ ^]^ At high concentrations of soluble gold in microbial systems, lysis and release of reductive biomolecules occur, providing supplementary resilience to the remaining biofilm.^[^ ^17j,k^ ^]^ This strategy could potentially be applicable to biomedical applications, wherein cytotoxic concentrations of ionic gold could be directly applied to a pathological site such as solid tumor. While the interior cells within the pathology may lyse, their released content would serve as a cocktail of reducing agents for formation of benign GNPs thus preventing gold ions from leaking out of the tumor at cytotoxic concentrations. However, if a direct delivery to the pathological site is not available then other strategies to control delivery and local concentration gold ions would need to be used.

### 8.5 Adding Control to GNP Biosynthesis

Although cellular driven biosynthesis might appear as a system “lacking” control, specificity of GNP biosynthesis could potentially be achieved through a number of approaches that can either adjust reaction kinetics in vitro or in vivo, or also through strategies to control delivery and release of gold ions.

#### 8.5.1 Controlling the Reaction Drivers of Biosynthesis

The major reaction drivers for GNP synthesis are similar to biological and benchtop strategies (temperature, concentration, etc.) and thus, approaches that can modify these reaction drivers could in turn influence particle synthesis.

It is well established that changes in the reaction temperature influences GNP formation;^[^ ^3a–c,f,h^ ^]^ and this is similarly reflected in works from biogeochemistry perspectives in microbial systems.^[^ ^17p^ ^]^ In mammalian cell gold biomineralization, Schwartz‐Duval et al.^[^ ^28b^ ^]^ demonstrated that an application of thermal energy via a laser illumination resulted in an increased Raman signal of ex vivo samples. They hypothesized that the increased Raman signal is associated with formation of larger gold particles, resulting from the additional thermal energy modifying the reaction kinetics of biosynthesis. Indeed, laser induced synthesis of gold particles from ions is documented elsewhere as well.^[^ ^58^ ^]^

Gold biomineralization by mammalian cells was also modified by the addition of prefabricated gold particles,^[^ ^28l^ ^]^ Na_2_SeO_3_,^[^ ^28j^ ^]^ FeCl_2_,^[^ ^28g,j^ ^]^ and graphene.^[^ ^28d^ ^]^ Further, these strategies also resulted in acquisition of novel properties such as nanoribboned morphology observed in the presence of prefabricated GNPs, which acted as scaffolds, that improved photothermal effect;^[^ ^28l^ ^]^ magnetic properties for MRI contrast in the presence of iron ions;^[^ ^28g,j^ ^]^ and approximately fourfold greater Raman signal from a co‐application of graphene compared to an application of gold ions alone.^[^ ^28d^ ^]^ Interestingly, *nuggets* of gold formed by microbes are often found not as pure gold but as mixtures with iron, copper, silver, or other metals,^[^ ^17o,q,x,aa^ ^]^ and the variety of minerals in microbial nanoparticle biosynthesis is vast.^[^ ^17a^ ^]^ From these observations, one could envision a scenario wherein other metal ions are supplemented alongside gold and the ratio of precursor metals is tuned for desirable optical or magnetic properties.

Tuning the concentrations of both gold ion and reductant determines the ensuing particles formed through benchtop synthesis,^[^ ^3a–c,f,h^ ^]^ and microbial precipitation.^[^ ^17v^ ^]^ Aside from externally delivered gold salts, other precursor components to gold biosynthesis are generated and maintained by the cells. Reports on mammalian cell biomineralization contain examples of modifications of the concentration of cellular reductants involved in gold biosynthesis. These studies used rotenone, ES936, and compound 968, to evaluate disruptions of gold particle biomineralization through the inhibition of NADH, QOH‐1, and glutamate, respectively, finding that these treatments resulted in reduction of fluorescent GNC formation.^[^ ^28f,h^ ^]^ But for biomedical applications, treatments to directly enhance particle formation at a pathological site would be even more desirable. An example of this approach was reported by Zhao et al.^[^ ^22a^ ^]^ where calcification of cell membranes was achieved in folate‐receptor overexpressing cancer cells. Their strategy relied on primary application of folic acid, which interacts with folate receptors on the membrane of cancer cells, followed by application of Ca^2+^ ions that resulted in a cancer specific mineralization and encapsulation of a calcium bioceramic on the cell surface, inducing cell death. This strategy could potentially be directly applied to enhance membrane‐templated GNP biosynthesis, as folic acid is known to have gold ion reducing capacity.^[^ ^59^ ^]^

#### 8.5.2 Controlled Release of Gold Ions and Targeted Biosynthesis

Strategies to control the release of gold ions could enable therapy with lower concentrations of gold, as well as systemic targeted applications aside from direct application to the pathological site. Only one of the current publications on mammalian gold biomineralization used a nano‐vectorized, albeit, a non‐targeted local delivery approach based on a PEG co‐aggregation with the gold ions.^[^ ^28b^ ^]^ Their PEG‐based strategy to package ions could potentially be adapted to include targeted moieties similarly to other PEG‐based nanovectors.^[^ ^60^ ^]^ Furthermore, there are many other hydrogel and nanoparticle strategies for controlled release of metal ions that could be adapted for gold ions.^[^ ^61^ ^]^ For example, the review by Janarthanan et al.^[^ ^61b^ ^]^ describes hydrogels loaded with Fe^3+^ and other ions including Al^3+^, Co^2+^, Ni^2+^, Cu^2+^, Zn^2+^, Zr^4+^, Ag^+^, La^3+^, Ce^3+^, Sm^3+^, Eu^3+^, Tb^3+^, Au^+^, Bi^3+^. Misra et al.^[^ ^61a^ ^]^ reported an ionic “OjoGel” loaded with Au^3+^ as a colorimetric sensing material to detect changes in the ascorbic acid content of tear films resulting from ocular injury. These hydrogel strategies can be adjusted to control the release of ions, as was shown in the report by Tian et al.,^[^ ^61g^ ^]^ where the degree of hydrogel cross‐linking was calibrated to tune the release of iron cations to kill bacteria; strategies like this could be applied to gold ions.

In addition, nanoparticle‐based strategies for delivering metal cations could also be adapted for delivering of gold ions.^[^ ^61c–f^ ^]^ The specific examples include: 1) Du et al.,^[^ ^61c^ ^]^ where Ag^+^, Cu^2+^, Zn^2+^, Mn^2+^, or Fe^2+^ were loaded into chitosan tripolyphosphate nanoparticles to enhance the antibacterial properties through the combination of chitosan and the cations; 2) Daza et al.,^[^ ^61d^ ^]^ who delivered Cs^2+^ ions using a Brij polymer based inverted micelle to inhibit cancer cell metabolism; 3) Tarn and Yu et al.,^[^ ^61e^ ^]^ who described the use of mesoporous silica nanoparticles loaded with Ca^2+^ ions to induce apoptosis in cancer cells; and 4) Zhu et al.,^[^ ^61f^ ^]^ who used nanodiamonds for controlled intracellular release of Cu^2+^, Ni^2+^, Cd^2+^, or Cr^3+^ cations to induce cell cytotoxicity.

An interesting strategy that involves direct modification of a biomineralization precursor was reported by Kim et al.^[^ ^62^ ^]^ In this study a mitochondria‐targeting moiety (i.e., cationic triphenylphosphonium) was conjugate with to an initiating precursor for silica biomineralization (i.e., trialkoxysilane). The authors showed that this conjugate specifically targeted cellular mitochondria resulting in aggregation of silica precursor moieties and a targeted mitochondrial silicification. A similar approach could be envisioned where gold ions are bound to a targeting moiety through a chelate for specific delivery to cellular targets in solution or inside a nanovehicle.

## 9 Summary of Translational Potential of GNP Biomineralization

We believe that the newly emergent field of GNP biomineralization in mammalian systems has potential to change the current paradigm of nanomedicine. By using pathological tissue to drive synthesis of therapeutic or diagnostic materials, this strategy is inherently personalized.

We see a strong potential for this process to significantly improve therapeutic interventions in dense biological tissues when applied at the pathological site. Key components of this treatment would include gold ion penetration through the dense tissue environment at the treatment site that would not be impeded by size, providing initial therapeutic cytotoxicity. As the gold treatment diffuses outward toward the periphery of the pathology, the concentration of the cytotoxic gold ions would decrease through conversion to benign but therapeutically activatable particles. A large portion of these particles would innately localize to cell nuclei, potentially providing a secondary mechanism for cytotoxic therapy through their disruption of intranuclear processes and, importantly, greatly enhancing efficacy of external interventions such as photothermal therapy.

In the cases when direct local delivery is not feasible, there are multiple available strategies that can enable systemic applications and can add targeted specificity to gold particle biosynthesis. However, realizing the potential of gold biomineralization in biomedical applications will require extensive mechanistic studies as many of the involved processes are largely unknown. Specifically, these unknowns include 1) the full mechanisms of GNP biosynthesis, 2) the timeframe of biosynthesis in different subcellular locations, and 3) how the formation of gold particles impacts the activity of the involved biomolecules. Making these discoveries would in turn better inform and enable targeted specificity for biomedical applications of particle biosynthesis as well as development of the appropriate disease specific applications.

## Conflict of Interest

The authors declare no conflict of interest.
